# Multifaceted roles of flavonoids mediating plant-microbe interactions

**DOI:** 10.1186/s40168-022-01420-x

**Published:** 2022-12-16

**Authors:** Lanxiang Wang, Moxian Chen, Pui-Ying Lam, Francisco Dini-Andreote, Lei Dai, Zhong Wei

**Affiliations:** 1 State Key Laboratory Breeding Base of Green Pesticide and Agricultural Bioengineering, Key Laboratory of Green Pesticide and Agricultural Bioengineering, Ministry of Education, Research and Development Center for Fine Chemicals, Guizhou University, Guiyang, China; 2 CAS Key Laboratory of Quantitative Engineering Biology, Shenzhen Institute of Synthetic Biology, Shenzhen Institutes of Advanced Technology, Chinese Academy of Sciences, Shenzhen, 518055 China; 3 Center for Crossover Education, Graduate School of Engineering Science, Akita University, Tegata Gakuen-machi 1-1, Akita City, Akita 010-8502 Japan; 4 Department of Plant Science & Huck Institutes of the Life Sciences, The Pennsylvania State University, University Park, PA USA; 5 Jiangsu Provincial Key Lab for Organic Solid Waste Utilization, National Engineering Research Center for Organic Fertilizers, Jiangsu Collaborative Innovation Center for Solid Organic Waste Resource Utilization, Nanjing Agricultural University, Nanjing, China

**Keywords:** Flavonoids, Host-microbe interaction, Microbiome, Root exudate, Rhizosphere

## Abstract

Plant-microbe interactions dynamically affect plant growth, health, and development. The mechanisms underpinning these associations are—to a large extent—mediated by specialized host-derived secondary metabolites. Flavonoids are one of the most studied classes of such metabolites, regulating both plant development and the interaction with commensal microbes. Here, we provide a comprehensive review of the multiple roles of flavonoids in mediating plant-microbe interactions. First, we briefly summarize the general aspects of flavonoid synthesis, transport, and exudation in plants. Then, we review the importance of flavonoids regulating plant-microbe interactions and dynamically influencing the overall community assembly of plant-root microbiomes. Last, we highlight potential knowledge gaps in our understanding of how flavonoids determine the interactions between plants and commensal microbes. Collectively, we advocate the importance of advancing research in this area toward innovative strategies to effectively manipulate plant-microbiome composition, in this case, via flavonoid production and exudation in plant roots.

Video Abstract

**Supplementary Information:**

The online version contains supplementary material available at 10.1186/s40168-022-01420-x.

## Introduction

Plant microbiomes greatly affect the host development, health, and ability to withstand biotic and abiotic stresses [1, 2]. The adaptation to distinct environmental conditions and responses to various stresses often trigger terrestrial plants to secrete chemical compounds through their root system into the surrounding environment (the rhizosphere). These sets of root exudates/chemicals have multiple roles in plant growth and stress responses, including the improvement of the rhizosphere soil chemical properties, their effects on enhancing plant nutrient uptake, and the recruitment of beneficial microbial taxa [3–7]. For example, amino acids and long-chain organic acids were shown to be secreted in Arabidopsis roots when plants were challenged with the foliar pathogen *Pseudomonas syringae* pv. tomato DC3000 (hereafter *Pst*. DC3000) [8]. These chemicals exhibited a remarkable modulation of the rhizosphere microbiome in subsequent plant generations, with an effect on disease suppression [8, 9]. In fact, several products from the plant secondary metabolism (PSM) have been shown to dynamically modulate the establishment of plant-associated microbiomes [10]. For example, flavonoids, terpenoids, strigolactones, and coumarins—all of which affect plant fitness and regulate the assembly of specific microbial taxa in the rhizosphere [11–14].

**Fig. 1:**
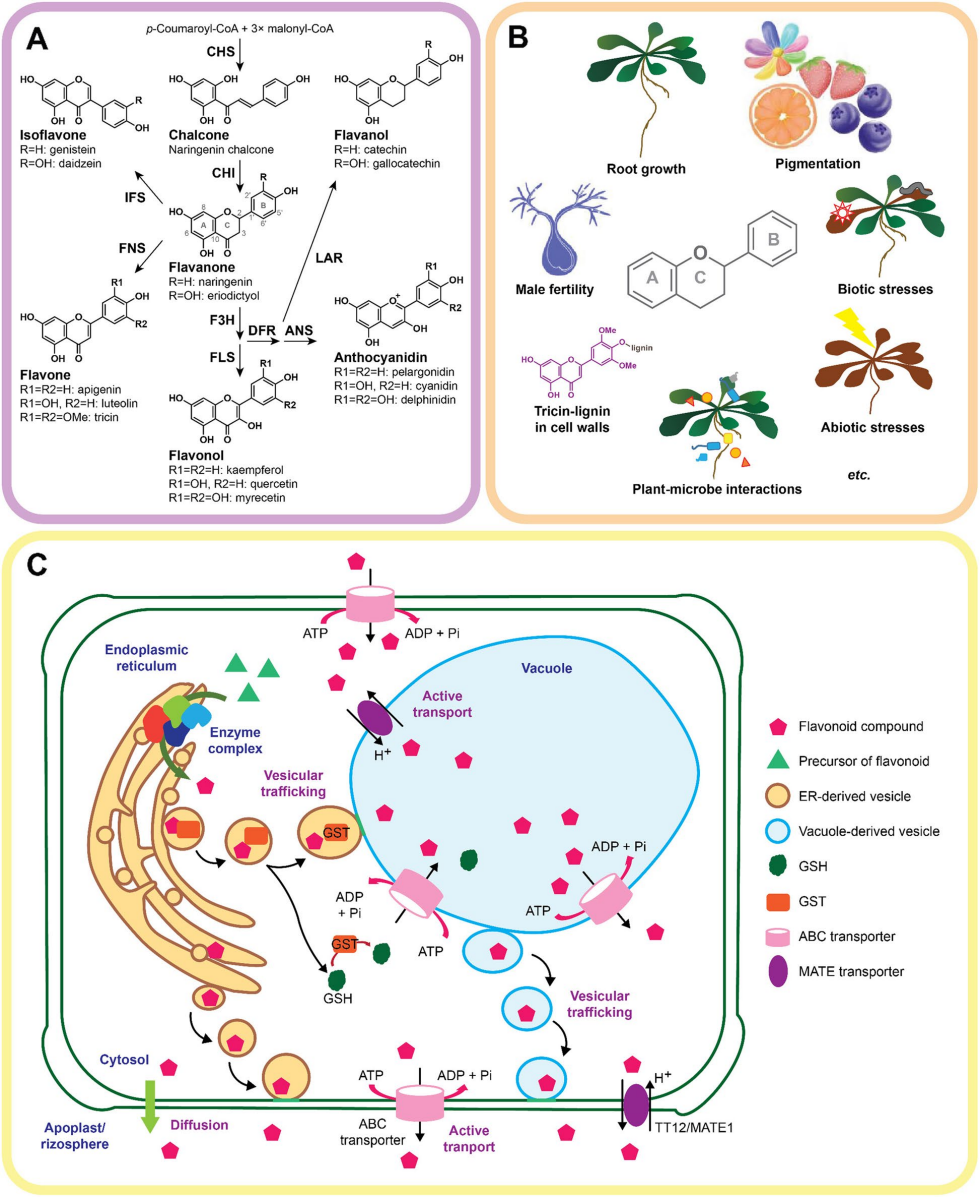
Flavonoid biosynthesis, function, and transport in plants. **A** Molecular structure of flavonoids, major subclasses of flavonoids, and the general flavonoid biosynthetic pathway in plants. **B** Biological functions of flavonoids in plants. **C** Intracellular and intercellular transport of flavonoids. CHS, chalcone synthase; CHI, chalcone isomerase; IFS, isoflavone synthase; FNS, flavone synthase; F3H, flavanone 3-hydroxylase; FLS, flavonol synthase; DFR, dihydroflavonol 4-reductase; LAR, leucoanthocyanidin reductase; ANS, anthocyanidin synthase; ABC, ATP-binding cassette; ADP, adenosine di-phosphate; ATP, adenosine tri-phosphate; Pi, phosphate; GSH, glutathione; GST, glutathione-S-transferase; MATE, multidrug and toxin efflux; TT12, transparent testa 12

Flavonoids constitute a major group of plant-specialized (or secondary) metabolites broadly studied in plant root exudates [6]. The term “flavonoids” commonly refer to a group of compounds that contain a diphenyl propane (C_6_-C_3_-C_6_) backbone in which two aromatic rings (A and B) are linked through the central three-carbon chain (C ring) (Fig. 1A). To date, more than 8000 flavonoid compounds have been isolated and identified from a diverse set of plant species [15]. And, the majority of these compounds are allocated within six major categories, namely flavonols, flavones, isoflavones, anthocyanins, flavanones, and flavanols. This classification is based on the saturation and oxidation of the C ring (Fig. 1A) [15]. Within each of these categories, flavonoid compounds differ by specific modifications in their molecules caused by the hydroxylation, methylation, glycosylation, or acylation of the A and B rings.

Flavonoids are essential molecules affecting plant growth and development, as well as acting on plant defense mechanisms against biotic and abiotic factors (Fig. 1B). They are involved in the determination of patterns of root growth by regulating the auxin polar transportation [16], controlling male fertility in maize (*Zea mays*), petunia (*Petunia hybrid*), tomato (*Solanum lycopersicum*), and rice (*Oryza sativa*), by affecting pollen germination and pollen tube growth [17–20]. Moreover, the compound tricin, a 3′,5′-dimethoxylated flavone, was reported as one of the “non-monolignol” lignin monomers incorporated into the cell wall lignin in the grass family [21–23]. Anthocyanidins—together with other co-pigments—are known to contribute to the red, purple, and blue coloration in most flowers, fruits, seeds, and vegetative tissues, thus attracting pollinators and influencing rates of plant seed dispersal by animals [24]. In addition, specific compounds from subgroups of flavones and isoflavones were shown to exhibit remarkable anti-fungal, weed-resistant, and insect-resistant activities in some crops [25–28]. Furthermore, mounting evidence suggests flavonoids to also be involved in light stress resistance [29–31], as well as in drought stress tolerance [32, 33]. However, our knowledge of how flavonoids mediate plant-microbe interactions and influence the overall microbiome assembly and functioning remains yet partially elusive. In this review, we summarize our current understanding of the fate of flavonoids by plants, as well as their roles in mediating dynamic plant-microbe interactions. We also discuss the extent to which flavonoids can impose changes in the assembly and functioning of the root microbiome and delve into the detailed mechanisms underpinning these processes.

### Biosynthesis and transport of flavonoids in plants and exudation

#### Flavonoids biosynthesis in plants

The core flavonoid biosynthetic pathway shared by land plants is initiated by the catalyzation of chalcone synthase (CHS) and chalcone isomerase (CHI). As essential enzymes involved in the biosynthesis of flavonoids, CHS and CHI are ubiquitous in flavonoid-producing plants. CHS and CHI are responsible for generating the middle C ring of the flavonoid skeleton that produces flavanones, such as naringenin (Fig. 1A). Flavanones serve as substrates for a range of downstream flavonoid biosynthetic enzymes, thus generating a diverse array of flavonoid metabolites. Depending on the types of downstream enzymes, different flavonoid molecules are formed and accumulated across different plant species (Fig. 1A). In dicot plants, flavanone 3-hydroxylase (F3H) and flavonol synthase (FLS) are required for the synthesis of flavonols and their derivatives accumulation. In some plant tissues that accumulate anthocyanidin, e.g., corolla and fruits, the activities of the enzymes dihydroflavonol 4-reductase (DFR) and anthocyanidin synthase (ANS) are required. In contrast, monocots are more prone to synthesize and accumulate flavones and their derivatives via flavone synthase (FNS). In addition, the isoflavone synthase (IFS) is uniquely present in legumes, which leads to the production of isoflavones. Besides, numerous enzymes that catalyze the hydroxylation, methylation, glycosylation, and acylation of flavonoids have been identified, which collectively contribute to the diversity of the flavonoids synthesized by plants [15]. Also of key importance, flavonoid biosynthetic enzymes can chemically interact with each other to form enzyme complexes. These are tethered to the cytosolic side of the endoplasmic reticulum (ER) via the ER-bound cytochrome P450 proteins [34] (Fig. 1C). The metabolon formed by these enzyme complexes is responsible for facilitating the channeling of the pathway intermediates that enhance the production of flavonoids [34]. Besides the ER, the tonoplast and nucleus are also considered the subcellular sites for flavonoid biosynthesis as some of the flavonoid biosynthetic enzymes are localized within these cellular compartments [35, 36].

### Transport of flavonoids in plants and exudation

In plants, flavonoids are transported within cells (Fig. 1C). In brief, after flavonoids are biosynthesized, these molecules are transported to vacuoles as major sites of storage. It is worth noticing that flavonoids can also be transported to other cellular compartments and even to the extracellular space. So far, three distinct mechanisms, namely vesicle trafficking, membrane transporters, and glutathione-S-transferase (GST)-mediated transport, have been proposed [37] (Fig. 1C). The vesicle trafficking of flavonoids was previously demonstrated in Arabidopsis *gfs9* (*green fluorescent seed*) mutants, enumerating the involvement of the GFS9 in membrane trafficking [38]. Also, two major types of membrane transporters, namely multidrug and toxin efflux (MATE) transporters and ATP-binding cassette (ABC) transporters, were described to be involved in the transport of flavonoids [39–41]. Besides, some studies suggested that GST is important for vesicle trafficking and membrane-mediated transport of flavonoids [42, 43]. However, whether these trafficking systems are associated with distinct localizations of flavonoids in plant tissues remains still to be explored.

Some flavonoids are also released to the root external environment [7, 44, 45], i.e., the plant rhizosphere (Fig. 1C). The discussed intracellular mechanisms of flavonoid transport and delivery are also associated with the release via root exudation [7]. In this scenario, the exudation of flavonoids in the rhizosphere has been proposed to be dynamically absorbed onto soil organic matter and/or to be rapidly degraded by soil-dwelling microbes [46, 47]. For instance, one study that simulated the daidzein (an isoflavone, Fig. 1A) distribution in the rhizosphere showed that this flavonoid compound can only be distributed a few millimeters away from the root surface, thus indicating that the soil mobility of daidzein is potentially greatly reduced by soil adsorption [48]. Given these interactions with soil physicochemical particles and as mediators of biotic interactions, several studies have been exploring the intricacies of flavonoid-mediated functions in the plant rhizosphere [49, 50].

### Flavonoids as mediators of plant-microbe interactions

Terrestrial plants are associated with a myriad of microbes establishing beneficial, detrimental, or commensal interactions. These dynamic interactions between host plants and microbes have strong impacts on plant growth, fitness, and even plant-microbe evolution [51]. Flavonoids exert important roles in mediating several of these interactions. Perhaps, mostly known for its importance in the process of Rhizobium nodulation in plants belonging to the *Fabaceae* family [52]. However, there are several other mechanisms by which flavonoids determine the interaction with other plant-associated microbes. From this section on, we will review some general and specialized roles of flavonoids in plant-microbe interactions, including pathogen, rhizobium, arbuscular mycorrhizal fungi (AMF), and other plant growth-promoting rhizobacteria (PGPR). Besides, we also included some examples of studies focusing on flavonoids as chemical cues associated with bacterial quorum sensing, thus mediating plant-microbe interactions.

### The role of flavonoids in plant-pathogen interaction

**Fig. 2:**
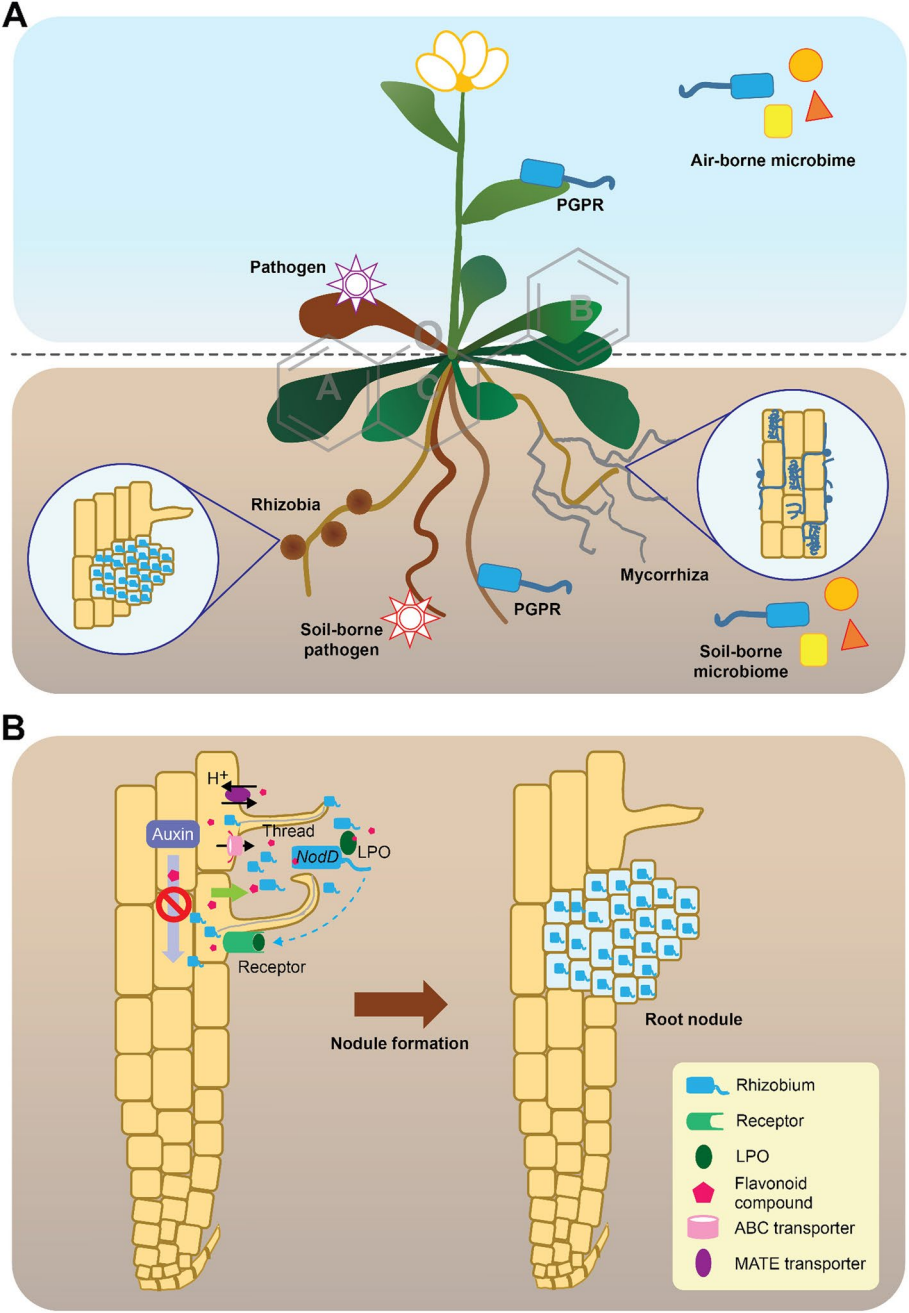
Multifaceted roles of flavonoids mediating plant-microbe interactions. **A** A conceptual overview of the distinct roles of flavonoids mediating plant-microbe interactions. **B** Illustration of flavonoids mediating nodule formation in plant roots. PGPR, plant growth-promoting rhizobacteria; LPO, lipo-oligosaccharide. Flavonoids are secreted into the rhizosphere via distinct mechanisms (see “Transport of flavonoids in plants and exudation” section). Specific flavonoid molecules in the rhizosphere are perceived by *Rhizobium*, which induces the expression of the *Nod* gene. This gene expression results in the synthesis and secretion of Nod factors (LPO). Legume plants recognize these Nod factors in the receptor and via the signal transduction pathway, thus eliciting a series of physiological and biochemical responses. Besides, flavonoid, such as kaempferol inhibits auxin transport, which enhances auxin concentration in the cortical cells and accelerates cell division and growth. Together, these physiological and biochemical processes lead to the formation of the root nodule

Several pathogen-induced flavonoid compounds are considered phytoalexins due to their accumulation in plant tissues upon pathogen infection, in addition to their antimicrobial activities (Fig. 2A). For example, the biosynthesis of sakuranetin (a flavanone) in rice was shown to increase the plant resistance to infection against (1) bakanae caused by *Fusarium fujikuroi* [53], (2) rice blast caused by *Magnaporthe Oryzae* [54], and (3) sheath blight caused by *Rhizoctonia solani* [55]. In sorghum (*Sorghum bicolor*), the pathogen-induced 3-deoxyanthocyanidins and luteolin (a flavone, Fig. 1A) exhibited remarkable toxicity to spores of the fungus *Colletotrichum sublineola*, the causal agent of anthracnose [56]. In addition, transgenic sorghum lines (*SbF3H1*) that produce flavonols and anthocyanidins were shown to enhance resistance against *C. sublineola* [57]. Also, metabolome and transcriptome analyses revealed that the accumulation of flavonoids can confer resistance to *Verticillium dahliae* in a spontaneous cotton (*Gossypium hirsutum*) mutant with red coloration [58]. Moreover, transgenic poplar (*Populus euphratica*) overexpressing two transcriptional factors, *PalbHLH1* and *PalMYB90*, had increased accumulation of proanthocyanidins, anthocyanins, and flavonols, that collectively provided resistance to infection by *Botrytis cinerea* and *Dothiorella gregaria* [59]. On the other hand, the reduced accumulation of flavonoids can enhance the susceptibility of transgenic plants to infection by pathogens. For example, MtABCG10-silenced composite Medicago (*Medicago truncatula*) was shown to be more sensitive to *Fusarium oxysporum*, a root-infecting pathogen. These authors suggested that isoflavonoid transport by MtABCG10 may have an essential role in pathogen defense [41]. Last, the knockdown of the transcriptional factor *HvWRKY23* in Fusarium head blight (FHB) resistant barley (*Hordeum vulgare*) was found to result in the downregulation of key flavonoid biosynthetic genes, as well as in the reduction of flavonoid accumulation, which collectively resulted in an increased disease incidence when challenged with the pathogen *Fusarium graminearum* [60]. In most of these examples, the mechanisms by which different flavonoids operate as antimicrobial compounds have been previously summarized [61]. In brief, these include microbial membrane lysis or rupture, inhibition of biofilm formation, cell envelope synthesis, nucleic acid synthesis, electron transport chain, and ATP synthesis, etc. (see [61] for additional details).

It is worth noticing that specific microbial taxa might have evolved resistance to flavonoid phytoalexins, thus enabling their survival and colonization of plant hosts. For example, the MexAB-OprM efflux pump was reported to mediate the resistance of *Pst*. DC3000 to flavonoids [62]. In a mutant line of *Pst*. DC3000 which lacks the MexAB-OprM transporter, flavonoids were shown to inhibit the function of the GacS/GacA two-component system that affects the synthesis of the microbial flagella and the type III secretion system of *Pst*. DC3000 [63]. Also, the multidrug transporter PSPTO_0820 and the multidrug resistance efflux pump outer membrane channel protein PSPTO_4977 in *Pst*. DC3000 were experimentally induced by flavanol catechin. Most interestingly, the knockout of these genes directly hindered the colonization of tomato roots by the *Pst*. DC3000 mutants [64]. Moreover, the HopZ1, a type III secreted effector of *P. syringae*, was found to physically interact with 2-hydroxyisoflavanone dehydratase (GmHID1). This was shown to result in an inhibition of daidzein production, thus promoting the proliferation of *P. syringae* in soybean (*Glycine max*) [65]. Last, microbial enzymes involved in the degradation of flavonoids have been reported to confer adaptive abilities to specialized pathogens to bypass flavonoid phytoalexins defenses. For example, a quercetin dioxygenase gene (QDO) was found in the genomes of several pathogens. This gene encodes an enzyme able to catalyze the cleavage of the flavonol carbon skeleton, which degrades flavonols into phloroglucinol carboxylic and phenolic acids [66, 67].

### The role of flavonoids in plant-rhizobium interaction

Flavonoids have been mostly studied with respect to their influence on rhizobium nodulation in *Fabaceae* plants (Fig. 2B). Early in vitro studies suggested that flavonoids serve as inducers of the expression of the rhizobia *nod* gene and as chemo-attractants enhancing the concentration of rhizobium on root surfaces. To illustrate these concepts, the exogenous addition of isoflavones was shown to induce the expression of *nod-lacZ* fusions in *Bradyrhizobium japonicum* [68]. Also, the imbibition of alfalfa (*Medicago sativa*) seeds induced the accumulation of quercetin-3-*O*-galactoside (a flavonol) and luteolin-7-*O*-glucoside (a flavone), which are molecules able to enhance the growth rate of *Rhizobium meliloti* [69]. And, the 5,7-dihydroxyl substitution of flavonoid compounds was reported to be primarily responsible for inducing the observed growth effect on rhizobium populations [69].

Recent developments in root hair transformation techniques allowed researchers to knock down flavonoid biosynthetic genes using RNAi to investigate the differential roles of flavonoids in root nodule formation. By silencing the *CHS* in Medicago, it was demonstrated that flavones are major inducers of *nod* genes in *Sinorhizobium meliloti* [70]. In addition, in vivo evidence further proved that flavones are essential for nodulation in Medicago—in this case—based on *FNS* knockout mutants [71]. In line with this, it was also shown that soybean *IFS* silenced mutants also proved the importance of isoflavones as inducers of *nod* gene expression in *B. japonicum* [70]. Additional analysis of root exudates from faba bean (*Vicia faba*) detected a total of six types of flavonoid compounds [72]. Within these, genistein (a isoflavone), hesperetin (a flavanone), and naringenin had significant correlations with nodule number and nodule dry weight [72]. All of these data suggest that flavonoids play a role in host specificity and selection of rhizobia strains for nodulation. Besides, auxin accumulation in cortical cells was also shown to be essential for nodule initiation, while flavonoids have a role in polar auxin transportation inhibition [73, 74]. Last, flavonoid accumulation during this process was demonstrated to be regulated by the CRE1-dependent cytokinin signaling [75].

Apart from the *Fabaceae*-rhizobium interaction, actinorhizal plants are known to form symbioses with nitrogen-fixing Actinobacteria taxa (also known as “actinomycetes”), in particular, those belonging to the genus *Frankia*. Despite the mechanisms underpinning these associations are yet not fully elucidated, one study has shown that flavonoids tend to accumulate inside the root nodules of *Casuarina glauca* [76]. Most interestingly, flavonoid-like compounds extracted from seeds of *Alnus rubra* were demonstrated to display both, nodulation induction and inhibition activities [77]. Also, two dihydrochalcone compounds extracted from the fruit of *Myrica gale* were shown to enhance growth and nitrogen fixation in the compatible *Frankia* strains [78]. In addition, flavonoid biosynthetic genes were activated in the root of the actinorhizal tropical tree *Alnus glutinosa* and *C. glauca* after inoculation with a *Frankia* bacterial strain [79]. Last, one study showed a reduction in nodule formation in flavonoid-deficient *C. glauca* root hairs and the rescue of nodule formation by the exogenous addition of naringenin [80].

### The role of flavonoids in plant-AMF interaction

Approximately 80% of land plants are able to establish symbiosis with arbuscular mycorrhizal fungi (AMF) (Fig. 2A) [81]. This symbiosis is one of the major interactions impacting plant fitness, particularly under phosphorus-limiting conditions [82]. Specific molecules in root exudates (in this case, those belonging to a class of plant hormones called “strigolactones”) are able to stimulate both AMF spore germination and germ tube branching [83]. In addition, secondary metabolites like flavonoids in root exudates are also dynamically involved in this process. For example, quercetin (a flavonol) in carrot (*Daucus carota*) root exudates was demonstrated to promote the hyphal growth of *Gigaspora margarita* [84]. This result corroborates with an in vitro assay showing that the exogenous amendment quercetin induces hyphal growth and hyphal branching of *Glomus etunicatum* [85]. Also, alfalfa seeds were shown to naturally secrete quercetin-3-*O*-galactoside, which stimulates the spore germination of *G. etunicatum* and *Glomus macrocarpum* [85]. Besides, quercetin secreted by the roots of the Chinese tallow tree (*Triadica sebifera*) was found to promote the colonization of roots by AMF [86]. Based on these collective findings, further studies should consider exploring the role of quercetin as a direct regulator of AMF establishment. For instance, the potential overexpression of quercetin in transgenic plants or the exogenous amendment of quercetin in prospective experimental systems can be used to potentially elucidate the mechanisms by which this flavonoid mediates AMF colonization.

On the other hand, the colonization of plant roots by AMF promotes plant growth and can significantly alter the production of plant secondary metabolites, including flavonoids. A study on olive (*Olea europaea*) tree roots demonstrated that AMF colonization increased the plant production of flavonoids and total phenols [87]. Likewise, heartsease (*Viola tricolor*) inoculated with *Rhizophagus irregularis* BEG144 was shown to produce higher levels of rutin (a flavonol) [88]. And, upon inoculation with the AMF strain *Glomus mosseae*, the pruning wastes of grapevine (*Vitis vinifera*) were found to produce larger amounts of quercetin [89]. This also aligns with a study showing the co-inoculation of *G. mosseae* and the bio-fertilizer Nitroxin in sorghum to significantly increase grain and protein yield, as well as total flavonoid content under severe drought stress [90].

### The role of flavonoids in plant-PGPR interaction

Plant growth-promoting rhizobacteria (PGPR) are microbes able to promote plant growth via direct or indirect mechanisms (Fig. 2A) [91]. Several studies have shown that flavonoids are active molecules mediating the communication between PGPRs and plants suggested that flavonoids might be playing a key role in these interactions. In addition, some studies have also reported the potential of specific microbial taxa to induce the expression and/or accumulation of flavonoids in plant tissues. For example, one study reported that the well-studied biocontrol PGPR *Bacillus subtilis* triggers flavonoid accumulation in tobacco and contribute to the suppression of the pathogen *Agrobacterium tumefaciens* [92]. Furthermore, flavonoids, like apigenin (a flavone) and phloretin (a chalcone), were shown to be sensed by *Pseudomonas fluorescens* 2P24 via the *Tet*R regulator *Phl*H. These flavonoids play an important role in enhancing the swarming motility and the production of cellulose and curli fibers in this non-symbiotic beneficial rhizobacterium. In addition, this mechanism of flavonoid sensing significantly contributes to the ability of this PGPR to move in the soil/root interface, thus favoring the successful colonization of the plant root surface [93].

Flavonoids are also involved in PGPR-mediated plant tolerance to abiotic stresses, especially salinity stress. For example, wheat (*Triticum aestivum*) inoculated with *Bacillus pumilus*, *Pseudomonas mendocina*, *Arthobacter* sp., *Halomonas* sp., and *Nitrinicola lacisaponensis* exhibited enhanced tolerance to salinity, in this case, an effect attributed to the accumulation of quercetin in response to the inoculation [94]. Also, the symbiotic plant growth-promoting actinobacterium *Glutamicibacter halophytocola* strain KLBMP5180 isolated from root tissues of the coastal halophyte *Limonium sinense* was shown to exert plant growth-promoting effects under salinity stress [95]. A study that integrated metabolomics and transcriptomics analyses demonstrated that flavonoids induced by the inoculation of KLBMP5180 are important in host salt stress tolerance [95]. It was also reported that root exudates of *L. sinense* attract PGPR taxa, such as *Bacillus flexus* KLBMP4941 under salt stress, which is associated with flavonoid accumulation and enhancement of plant growth [96]. Moreover, the inoculation of *Azospirillum lipoferum* FK1 in salt-treated chickpea (*Cicer arietinum* L.) was demonstrated to significantly promote plant growth via increased flavonoid production [97]. Worth mentioning, similar results were also reported in NaCl-treated soybean inoculated with *Bacillus firmus* SW5 [98].

Flavonoids have also been shown to contribute to plant tolerance to water and nutrient deficiencies via PGPR recruitment and microbial activities. For example, the inoculation of drought-treated pennyroyal (*Mentha pulegium* L.) with *Azotobacter chroococcum* and *Azosporollum brasilense* enhanced the total flavonoid accumulation and improved plant growth [99]. Recently, the bacterium *Aeromonas* sp. H1 was reported to enhance the dehydration resistance of Arabidopsis via specific flavonoid accumulation [100]. Also, the presence of *Steotrophomonas maltophilia* enhanced the total flavonoid content in peanut (*Arachis hypogea*), in this case, under nitrogen-limiting conditions [101]. And, root-derived flavones, such as apigenin and luteolin, were demonstrated to promote the enrichment of Oxalobacteraceae taxa associated with maize growth under nitrogen-limiting conditions [102]. Moreover, apigenin and luteolin in the exudates of rice plants were shown to trigger biofilm formation of *Gluconacetobacter diazotrophicus*, thus enhancing the potential of root surface colonization and improving biological nitrogen fixation under nutrient-limiting soil conditions [103].

Despite that we still lack a full appreciation of the specificity of diverse flavonoid molecules mediating the interaction between plants and PGPRs, some studies have shown that the inoculation with a single PGPR strain is able to induce flavonoid accumulation in plants. For example, after the inoculation with *Paenibacillus pabuli* strain P7S, the biomass and anthocyanin production in Arabidopsis were found to increase [104]. Also, *Eucalyptus grandis* “treated” with *Streptomyces* PM9 displayed higher levels of flavonoids in shoots, albeit similar values were reported in root tissues [105]. Last, an elevated flavonoid accumulation was also detected in grapevine leaves and roots inoculated with *Paraburkholderia phytofirmans* PsJN [106]. Thus, taken together, these results illustrate that PGPR inoculation may improve host plant performance—to some extent—by mediating the dynamic flavonoid accumulation that varies across distinct contexts and environmental conditions.

### The role of flavonoids in bacterial quorum sensing

Quorum sensing is a bacterial cell-to-cell communication process triggered by auto-inducers (AIs) signaling molecules once cell density reaches a certain threshold. Quorum sensing mediates a series of bacterial genes expression associated with important functions, such as the production of enzymes, secretion of exopolysaccharides and toxins, the establishment of biofilm, swarming motility, and horizontal gene transfer [107]. The class of molecules of *N*-acyl homoserine lactones (AHLs) is the most studied AIs present in Gram-negative bacteria. Most interestingly, some specific flavonoid molecules can be further transformed into AHL types. For example, naringenin (a flavanone), daidzein and genistein (isoflavones) in the rhizosphere were shown to be broken down by *Rhizobium* species to form *p*-coumaric acid [108]. And, in a follow-up step, *p*-coumaric acid can be transformed into *p*-coumaroyl-homoserine lactone (a type of AHL) by the photosynthetic bacterium *Rhodopseudomonas palustris* [109]. In addition, the production of AHL molecules in three rhizobia species (*Sinorhizobium fredii* SMH12, *Rhizobium etli* ISP42, and *Rhizobium sullae* IS123) was shown to be enhanced when these strains were cultured in the presence of specific flavonoids (*nod* gene inducer) [110]. On the other hand, Medicago plants inoculated with rhizobia-producing AHLs were also shown to enhance the expression of genes associated with flavonoid biosynthesis [111]. Albeit the significance of this study, the opposite effect has also been reported. For example, it was shown that barley plants produced a lower amount of flavonoids when inoculated with the AHL-deficient mutant of *Acidovorax radices* N35 compared with wild-type *A. radices* N35 [112].

Flavonoids can also have a direct effect on strain-specific bacterial quorum sensing. For example, one study showed that flavonoid compounds can inhibit biofilm formation in *Pseudomonas aeruginosa* via effects in the AI-binding receptors, namely LasR and RhlR [113]. Further, two hydroxyl moieties in the A-ring backbone were shown to be essential for this inhibition activity [113]. Also, naringenin was found to be effective in inhibiting quorum-sensing response in *P. aeruginosa* via binding directly to the LasR only at low concentrations of AIs [114]. This effect of quorum-sensing suppression in *P. aeruginosa* was also shown to result in reduced production of virulence factors (i.e., pyocyanin and elastase) [114, 115]. Last, similar results have also been reported in other pathogens, such as *Staphylococcus epidermidis* [116]. Thus, it is possible to speculate that flavonoids might likely be a yet-underexplored class of molecules potentially associated with pathogen control, for instance, via quorum-sensing suppression.

### Multifaceted roles of flavonoids mediating the assembly and functioning of root microbiomes

Several products of PSM in root exudates—including phytohormones and diverse flavonoid molecules—dynamically regulate the recruitment and functioning of microbial taxa in the rhizosphere [49, 117, 118]. For example, a study showed the importance of triterpenes (another class of PSM found in Arabidopsis root exudates) in structuring the plant root microbiome [12]. In fact, most flavonoid molecules can act as signals for the recruitment of specific microbial taxa and/or act as substrates for microbial growth, thus linking their production and consumption with the recruitment in the plant rhizosphere [100]. In addition, some flavonoids can also have a negative effect on taxa recruitment, for instance, via anti-microbial activities [48, 117]. Although some of these aspects were covered in the previous sections of this article, herewith we provide a more holistic perspective on how flavonoids can be seen as underlying regulatory molecules in plant exudates that influence the overall root microbiome recruitment and functioning.

### Flavonoids modulate dynamic changes in rhizosphere microbiomes

Due to the importance of dietary intake of flavonoids influencing human health, interactions between flavonoids and microbiomes are particularly well described in the human and (model) animal gut systems. Similar to the plant rhizosphere, the animal gut also has a dynamic gradient of nutrients that provides multiple resources and ecological niches for microbial colonization. Albeit major differences exist between these two systems, concepts derived from one system can easily be interpreted and translated into the other. The most striking difference between these systems relies on the distinct concentrations of oxygen in these habitats and how it affects microbial taxa recruitment and chemical reactions in the system. Some microbes in the human gut can consume, modify, or degrade flavonoids. For example, a novel ene-reductase that initiates the degradation of flavones and flavonols, namely flavone reductase (FLR), was discovered in the gut bacterium *Flavonifractor plautii* ATCC 49531 [119]. Besides, it was shown that strains belonging to *Lactococcus* and *Enterococcus* isolated from human fecal samples have de-glycosylation activities for specific *C*- and *O*-glycosides of flavonoids [120, 121]. These authors also reported that the bacterium *Blautia* sp. MRG-PMF1 is able to demethylate multiple polymethoxyflavones [122]. In addition, flavonoids ingested in the diet can affect patterns of gastrointestinal microbial community assembly [123, 124]. For example, as one of the primary components in multiple plant extracts (e.g., cucumber, carrot, and broccoli seed flours), kaempferol-3-*O*-rutinoside (a flavonol) was reported to promote an increase in the diversity of the gut microbial community, possibly through their scavenging capacity of reactive oxygen species [125]. Also, as an important active flavonoid derivative, quercetin-3-*O*-glucuronide (a flavonol) was found to partially restore the dysbiosis of the gut microbiome (i.e., affecting the Bacteroidetes and Firmicute balance). In addition, the administration of quercetin was reported to improve the gut microbial diversity in mice that were previously treated with antibiotics [126]. Also, another study highlights the link between the anti-inflammatory property of quercetin and the gut microbiome in mice models used to study inflammatory bowel disease. Specifically, the abundances of *Lactobacillus*, *Bacteroidetes*, and *Bifidobacterium* were reported to increase, whereas the abundances of *Fusobacterium* and *Enterococcus* decreased by quercetin pre-administration [127]. In an example based on a non-model mammal system, a total of 97 flavonoids were identified in bamboo, of which more than 70% were able to be metabolized by the panda gut microbiome. These were further demonstrated to negatively affect microbial diversity by enriching the abundance of cellulose-degrading microbes [128].

Particularly in the soil and plant rhizosphere, it was shown that soils treated with daidzein—an important flavonoid found in Medicago root exudates—had a significant negative effect on α-diversity (i.e., for bacterial community), suggesting a potential impact on specific soil microbial populations [48]. Similarly, the α-diversity of a soil microbiome treated with quercetin was also reduced, albeit this treatment resulted in the positive growth of specific (and potentially beneficial) taxa [117]. In detail, the soil treated with daidzein led to an increase in the relative abundance of Comamonadaceae [48], while the soil treated with quercetin resulted in a relative enrichment of *Pseudarthrobacter*, in addition to a higher overall relative abundance of Proteobacteria [117]. Also, a study on maize root exudates showed flavone compounds, such as apigenin and luteolin, to enhance the abundance of Oxalobacteraceae in the plant rhizosphere. In line with that, microbial isolates within the *Massilia* genus (also Oxalobacteraceae) are known to improve host performance under nutrient-limiting conditions [102]. Last, it is important to point out that properly studying flavonoids in soil systems is often challenging, mostly due to the chemical interactions of these molecules with other soil particles. For example, it was shown that dissolved organic carbon in soil can significantly reduce the lifetime of flavonoids, which, in turn, directly affects the signaling communication mediated by these molecules in the system [47].

### Differential responses of microbial taxa to plant-derived flavonoids

Flavonoids as complex signaling molecules can be perceived, consumed, and modified by different microbial taxa. Also, the specificity of these molecules and chemical dynamics can differentially influence taxa abundances and the overall microbiome composition [102]. As mentioned above, the de-glycosylation of *O*- and *C*-glycosides of flavonoids performed by gut microbes is an example of gut-specialized taxa capable of hydrolyzing flavonoid glycosides. Also, flavonoid aglycones released in the gut were shown to be degraded into small molecules by specialized microbial enzymes [119–121]. Conversely, similar enzymatic processing of flavonoids has been reported in the rhizosphere [117]. Mounting evidence has been suggesting specific microbial taxa, such as *Rhizobia* and *Pseudomonas*, to actively degrade flavonoid molecules [46, 108]. These molecules are used as energy sources by specialized microbes directly via the degradation of the carbon skeleton of flavonoids and further channeling of by-product degradation into the tricarboxylic acid cycle [46].

As an additional mechanism of microbiome modulation, molecules derived from the degradation of flavonoid compounds (e.g., phenolic acids) present in root exudates can also affect the rhizosphere microbiome [129]. In addition, these derived molecules often exhibit higher antioxidant activities than the precursor molecules [130], which may not only affect the specialized taxa but the overall micro-scale environment in the plant rhizosphere. Last, it is tempting to speculate that since a similar molecule can often have opposite effects (positive or negative) on distinct microbial taxa, the production, perception, and utilization of different flavonoids might likely be associated with eco-evolutionary dynamics of the host with distinct microbial taxa in the system, thus serving as a resource that triggers species interactions. In this sense, microbes may likely compete with each other in a direct or indirect manner [131], which may contribute to the dynamic structure and functioning of microbiomes.

## Conclusions and future perspectives

Flavonoids are plant metabolites with a variety of functions in both eukaryotic and prokaryotic organisms. Although the biosynthesis and metabolisms of flavonoids in plants have been broadly studied, the multifaceted roles of these compounds mediating host-microbe interactions remain largely unexplored. This includes their dynamic overall effects on community assembly and the molecular mechanisms by which these molecules act as chemical signals in the plant rhizosphere. Given the current need to harness specific microbial taxa and functions toward the development of sustainable practices in agroecosystems [132], better understanding the influences of flavonoids and their derivatives on plant commensal taxa has fundamental implications. In this sense, different and complementary focuses are emerging in this field, including the sensing mechanisms of flavonoids by microbes and their signal transduction in the cells [133], as well as the ecological implications of flavonoids as mediators of host-microbe interactions [102]. Of key importance, several plant mutant lines are currently available to investigate the potential roles of specific flavonoid molecules in rhizosphere biology [134]. These plant mutant libraries can be used to explore how distinct root exudates containing distinct flavonoid compositions and concentrations dynamically affect the recruitment and functioning of microbial taxa in the rhizosphere. As a follow-up, the modulation of specific microbial populations via flavonoid metabolism to either include the loss-of-function or gain-of-function in the system might emerge as a promising approach to effectively engineer plant-beneficial microbiomes.

## Data Availability

Data sharing not applicable to this article as no datasets were generated or analyzed during the current study.
